# When should bladder cancer prompt evaluation for Lynch syndrome? An evidence-informed clinical framework

**DOI:** 10.3389/fonc.2026.2015634

**Published:** 2026-10-28

**Authors:** Nicola Fazaa, Maya Gofer, Etan Eigner, Melissa Atallah, Yuval Shafran, Gilad Amiel, Azik Hoffman

**Affiliations:** Department of Urology, Rambam Health Care Campus, Haifa, Israel

**Keywords:** bladder cancer, Lynch syndrome, mismatch repair deficiency, MSH2, urothelial carcinoma

## Abstract

Lynch syndrome (LS) is an established cause of urothelial cancer, yet hereditary evaluation remains far better defined for upper tract urothelial carcinoma than for bladder cancer. The clinically relevant question is not whether bladder cancer belongs to the LS spectrum, but when a bladder cancer diagnosis should prompt evaluation for previously unrecognized LS. This mini-review examines the clinical and molecular findings that may justify hereditary assessment in patients presenting with bladder cancer and proposes an evidence-informed framework for their interpretation. The strongest signals include a known familial LS variant, a personal or family history of LS-associated malignancy, and tumor evidence of mismatch repair deficiency, microsatellite instability, or a pathogenic MMR alteration. Previous or synchronous upper tract disease and younger age may increase suspicion, whereas tumor grade alone is insufficiently specific. Tumor testing should guide, but not replace, germline evaluation, and negative tumor findings do not fully exclude hereditary disease when clinical suspicion remains high. Confirmation of LS has clear implications for lifelong cancer prevention and cascade testing, while its effect on bladder cancer treatment and urologic surveillance remains uncertain. Prospective bladder-specific studies are needed to validate testing strategies and define clinical actionability.

## Introduction

Lynch syndrome (LS) is a common autosomal dominant cancer predisposition syndrome caused by germline pathogenic variants in the mismatch repair (MMR) genes *MLH1, MSH2, MSH6*, and *PMS2*, or by deletions involving *EPCAM* that lead to epigenetic silencing of *MSH2* (1, 2). LS is estimated to affect approximately 1 in 279 individuals and is associated with a broad spectrum of malignancies, including urothelial cancer (1, 2). Loss of MMR function leads to the accumulation of replication errors and may result in microsatellite instability (MSI), a characteristic molecular feature of many LS-associated tumors (2). Urothelial malignancies are particularly relevant among *MSH2* carriers, who have the strongest documented association with urinary tract cancer among the major MMR gene carriers (3–5). In the Prospective Lynch Syndrome Database, urothelial cancer accounted for 19 deaths among *MSH2* carriers undergoing surveillance, compared with 20 deaths from colon cancer, highlighting its clinical relevance in this population (6).

Upper tract urothelial carcinoma (UTUC) is a well-recognized LS-associated malignancy, with dedicated recommendations for hereditary evaluation that include assessment of personal and family history, tumor MMR/MSI testing, genetic counselling, and germline testing (7, 8). Bladder cancer (BC) is also associated with LS, particularly among *MSH2* carriers, yet current bladder cancer guidance does not provide a comparable Lynch-specific testing pathway (4, 5, 9, 10).

LS may present with BC in the absence of UTUC, and recent germline testing data have identified *MSH2* carriers whose urothelial malignancy was confined to the bladder (11). This raises a practical question for the urologist: which patients presenting with bladder cancer should prompt evaluation for underlying Lynch syndrome? Current evidence does not provide a validated bladder-specific answer (9, 10). This mini-review therefore focuses on the clinical and molecular features that may help identify patients who warrant tumor-based assessment, genetic counselling, and germline testing, while highlighting areas in which evidence remains insufficient.

## The testing gap between UTUC and bladder cancer

The association between Lynch syndrome and UTUC is sufficiently established to have been incorporated into contemporary hereditary evaluation pathways (7, 8). Current EAU guidance recommends assessment of personal and family cancer history, referral for germline testing when hereditary UTUC is suspected, and consideration of tumor MMR or MSI testing even in the absence of an obvious hereditary history (8). The AUA/SUO takes a broader approach, recommending routine tumor-based MMR or MSI testing in UTUC, with genetic counselling and germline evaluation when the findings raise concern for Lynch syndrome (7).

This difference in approach is supported by the substantially higher prevalence of MMR abnormalities in UTUC compared with bladder cancer. In a systematic review and meta-analysis, dMMR was identified in 8.95% of UTUC and 2.30% of bladder cancers, while MSI-H was reported in 8.36% and 2.11%, respectively (12). Even in UTUC, however, clinical and tumor-based selection strategies remain imperfect. In a recent prospective cohort in which germline testing was offered irrespective of clinical suspicion, pathogenic MMR variants associated with Lynch syndrome were identified in 3.1% of tested patients, including carriers who would not have been identified by all conventional selection criteria (13).

Bladder cancer presents a less clearly defined situation. Although it is a recognized Lynch syndrome-associated malignancy, current bladder cancer guidelines do not provide a dedicated pathway for deciding which patients should undergo MMR/MSI testing or germline evaluation (9, 10). The lower prevalence of dMMR and MSI-H makes direct adoption of the UTUC strategy difficult to justify, yet it remains uncertain whether a predominantly history-based approach is sufficient to identify affected patients (10, 12). This leaves the central practical question: which patients presenting with bladder cancer should prompt further evaluation for Lynch syndrome, and which clinical or molecular findings are sufficiently meaningful to trigger that process?

Routine tumor-based MMR assessment is incorporated into contemporary colorectal and endometrial cancer pathways, but an equivalent strategy has not been established for bladder cancer (14, 15). In bladder cancer, dMMR/MSI-H is relatively uncommon, and prospective bladder-specific evidence supporting universal screening is lacking (12). On the basis of current evidence, we therefore favor a selective approach rather than universal MMR/MSI testing, integrating personal and family history with available tumor molecular findings. MMR IHC and/or MSI testing can provide a useful route to hereditary evaluation, but a negative tumor screen should not override strong clinical suspicion for Lynch syndrome (10, 16).

## Who should be evaluated for Lynch syndrome and how?

No validated bladder cancer-specific criteria currently define which patients should undergo evaluation for Lynch syndrome (10). Rather than treating individual clinical features as testing criteria, the available evidence is better interpreted as a hierarchy of signals that differ in their strength and bladder-specific support.

A known familial pathogenic MMR variant provides the most direct indication for hereditary evaluation. Family history is also important, but the relevant history extends well beyond urothelial cancer. In a recent germline testing registry, only 22% of *MSH2* carriers with urothelial cancer reported a family history of bladder cancer or UTUC, compared with 80% reporting a family history of colon cancer and 31% endometrial cancer (11). Thus, a family history focused only on urinary tract malignancies may fail to identify an otherwise suggestive Lynch syndrome pedigree.

A personal history of another Lynch-associated malignancy should similarly increase suspicion. Prior nonurothelial malignancies were reported in 54–63% of MMR variant carriers in the same registry, although these figures derive from a population selected for germline testing and should not be extrapolated to unselected patients with bladder cancer. The practical point is that the bladder tumor should not be considered in isolation from the patient’s broader cancer history (10, 11).

The absence of UTUC should not be reassuring by itself. Although MMR variants were more strongly enriched among patients with UTUC, 58% of *MSH2* carriers in the registry had bladder-only urothelial disease (11). A previous or synchronous UTUC remains an important hereditary clue, but requiring upper tract involvement would miss a substantial proportion of *MSH2* carriers presenting with bladder cancer alone.

Age at diagnosis is a less reliable discriminator. In the series by van der Post et al., bladder cancer in Lynch syndrome families was diagnosed at a mean age of approximately 60 years, about a decade younger than sporadic bladder cancer in the background population (3). However, Lynch-associated urothelial cancer spans a wide age range, and genotype may influence the age at presentation; in a recent LS-UTUC cohort, *MSH6* carriers presented at a median age of 68.5 years compared with 58 years for *MSH2* carriers (17). Younger age may therefore strengthen suspicion, but no bladder-specific age threshold has been validated, and UTUC-derived cutoffs should not simply be transferred to bladder cancer (10, 17). Although smoking is a major established risk factor for bladder cancer (9), current evidence does not support using smoking status to select patients for Lynch syndrome evaluation.

Tumor grade is also insufficient as a Lynch-specific trigger. Pietzak et al. found pathogenic or likely pathogenic germline cancer-predisposition variants in 13.5% of patients with high-grade NMIBC and none among those with low-grade disease, but most identified variants involved DNA repair pathways other than MMR (18). High-grade disease may therefore identify a population with greater overall germline susceptibility, but current evidence does not support using grade alone to select patients for Lynch syndrome evaluation (10, 18).

Tumor findings provide a second route to hereditary evaluation. dMMR or MSI-H is an important molecular clue, but neither finding establishes Lynch syndrome, because the same phenotype can result from acquired somatic MMR alterations (19). Conversely, absence of demonstrable MMR deficiency in a bladder tumor does not completely exclude an underlying germline syndrome.

This limitation is particularly relevant in urothelial cancer. Rasmussen et al. found loss of MMR protein expression in 88.7% of 97 urothelial tumors from Lynch syndrome carriers and their first-degree relatives, but concordance with MSI varied according to the assay used (16). Retained MMR expression was disproportionately observed in lower-tract tumors; among confirmed carriers, four of the five tumors with retained MMR expression arose in the lower urinary tract. These findings argue against using a negative tumor screen as an absolute rule-out when the personal or family history remains strongly suggestive.

The pattern of MMR protein loss may also be informative. Loss involving MSH2/MSH6 is particularly relevant in urothelial cancer given the strong association between germline *MSH2* variants and Lynch-associated urothelial malignancy, although abnormal immunohistochemistry still requires germline confirmation (3, 16, 19).

Tumor next-generation sequencing can provide another entry point when a pathogenic MMR alteration is identified during routine molecular profiling. Such a result cannot by itself determine whether the alteration is inherited or somatic, and tumor-only sequencing may fail to detect pathogenic germline MMR variants (20). An MMR alteration identified on tumor sequencing should therefore prompt consideration of germline evaluation rather than be interpreted as a diagnosis of Lynch syndrome (10, 20).

From a clinical standpoint, the clearest reasons to pursue Lynch syndrome evaluation in a patient with bladder cancer are a known familial pathogenic variant, a convincing personal or family history of Lynch-associated malignancy, and tumor evidence of MMR dysfunction or a pathogenic MMR alteration. Previous or synchronous UTUC and younger age may further raise suspicion, whereas tumor grade alone is not sufficiently specific to serve as a Lynch syndrome trigger. These distinctions are based on the available evidence rather than on a validated bladder cancer-specific model, since most published data derive from known Lynch syndrome carriers, selected germline testing cohorts, or mixed upper- and lower-tract urothelial cancer populations rather than prospectively tested, unselected bladder cancer cohorts (10, 11, 16). The relative clinical weight, interpretation, and practical implications of these findings are summarized in **Table 1**.

**Table 1:** Clinical and molecular features relevant to Lynch syndrome evaluation in bladder cancer.

Finding	Interpretation in bladder cancer	Practical implication	Key references
Known familial LS pathogenic/likely pathogenic variant	Direct evidence of familial hereditary risk	Targeted germline evaluation for the known familial variant	(23)
Personal history of another LS-associated malignancy	Strong clinical signal for an underlying hereditary syndrome	Supports hereditary evaluation	(11, 23)
Family history suggestive of LS	Relevant family history extends beyond urothelial cancer and should include the broader LS tumor spectrum	Strong reason to consider hereditary evaluation	(11)
Tumor dMMR or MSI-H	Important molecular clue, but does not establish LS	Should prompt consideration of germline evaluation	(12, 16, 19)
Pathogenic/likely pathogenic MMR alteration on tumor NGS	Tumor-only sequencing cannot reliably establish germline origin	Should prompt germline evaluation	(20)
Previous or synchronous UTUC	Increases suspicion for LS, although absence of UTUC does not exclude a bladder-only presentation	Supportive clinical feature	(3, 4, 11)
Younger age at bladder cancer diagnosis	May increase suspicion, but no validated bladder-specific age cutoff exists	Supportive modifier; should not be used alone	(3, 17)
High-grade bladder cancer	Enriches for germline cancer-predisposition variants overall, but available data do not show LS-specific enrichment	Insufficient as a stand-alone LS trigger	(18)

LS, Lynch syndrome; UTUC, upper tract urothelial carcinoma; dMMR, mismatch repair deficiency; MSI-H, microsatellite instability-high; NGS, next-generation sequencing.

Evaluation should separate two distinct questions: whether the bladder tumor shows evidence of MMR deficiency, and whether the patient carries an inherited Lynch syndrome-associated alteration (16, 19). MMR immunohistochemistry assesses expression of MLH1, MSH2, MSH6, and PMS2, whereas MSI testing identifies the genomic instability resulting from defective mismatch repair (19). These approaches are complementary but not fully interchangeable in urothelial cancer. In the series by Rasmussen et al., concordance between MMR immunohistochemistry and MSI ranged from 70.6% with a conventional MSI assay to 90.3% with a sequencing-based 54-marker assay (16).

Tumor testing should not be used as an absolute gatekeeper to germline evaluation. Retained MMR protein expression and microsatellite-stable tumors have been observed in patients with germline MMR variants, including in Lynch syndrome-associated urothelial tumors (16, 19). A patient with a known familial pathogenic variant or a strongly suggestive personal or family history may therefore still warrant germline evaluation despite a negative tumor screen. Germline testing establishes Lynch syndrome by identifying a pathogenic or likely pathogenic inherited alteration in an LS-associated MMR gene, or a pathogenic *EPCAM* deletion resulting in *MSH2* silencing (10, 19). Hereditary assessment should also account for contemporary multigene germline testing. When no familial LS variant is known, broader panels may identify pathogenic variants beyond the MMR genes, as reported in selected bladder and urothelial cancer cohorts (10, 11, 18). However, these data are insufficient to support universal multigene germline testing for all patients with bladder cancer. Panel selection should therefore remain guided by personal and family history, tumor findings, and genetic counselling.

A dMMR or MSI-H tumor with negative germline testing presents a different diagnostic problem. Tumor MMR deficiency is not specific to Lynch syndrome, and biallelic somatic alterations in MMR genes can produce the same phenotype in the absence of an inherited defect (19, 21). In unresolved cases, additional tumor sequencing or paired tumor–germline analysis can help identify a somatic explanation for MMR deficiency and distinguish it from an inherited syndrome. The evidence supporting this approach, however, comes predominantly from Lynch syndrome diagnostics in other tumor types, and bladder-specific data remain limited (10, 21).

Tumor next-generation sequencing provides another route to hereditary evaluation when an MMR alteration is detected during molecular profiling, but tumor-only sequencing cannot reliably determine whether such an alteration is germline or somatic (20). In a pan-cancer cohort of more than 21,000 patients, tumor-only sequencing failed to detect 18.8% of pathogenic germline variants in MMR genes (20). A pathogenic MMR alteration identified in the tumor may therefore prompt germline evaluation, whereas a negative tumor-only result should not be used to exclude Lynch syndrome when the clinical context remains suggestive (10, 20). A practical, evidence-informed framework integrating these clinical and molecular pathways is summarized in **Figure 1**.

**Figure 1:**
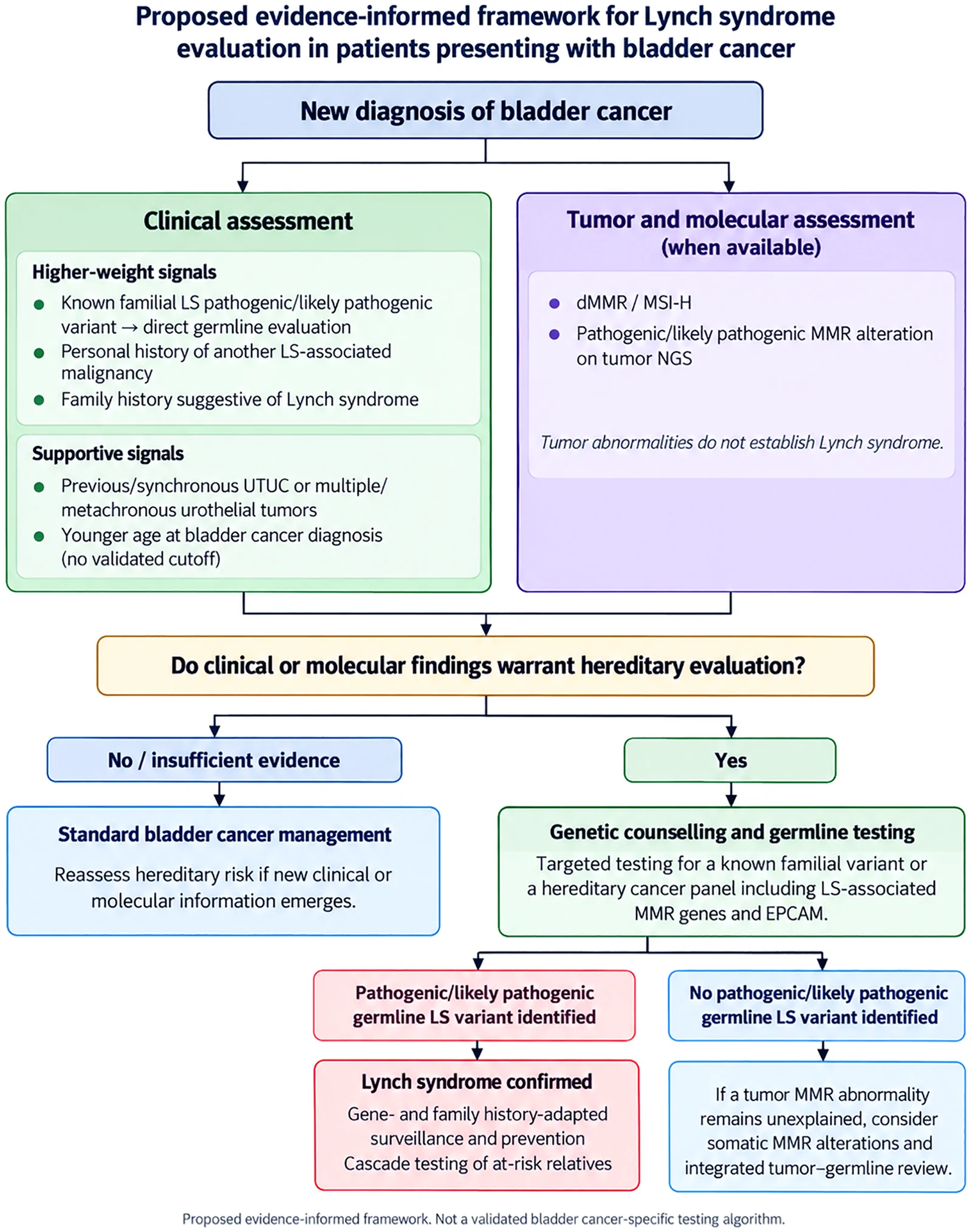
Author-proposed evidence-informed framework for Lynch syndrome evaluation in patients presenting with bladder cancer. The framework integrates clinical history and tumor molecular findings according to the relative strength of the available evidence. Known familial Lynch syndrome variants, personal or family history of Lynch-associated malignancy, and tumor MMR abnormalities represent the strongest signals for hereditary evaluation, whereas younger age and previous or synchronous urothelial malignancy are supportive rather than stand-alone criteria. Tumor dMMR/MSI-H or pathogenic MMR alterations do not establish Lynch syndrome and require germline confirmation. When germline testing is negative despite an unexplained tumor MMR abnormality, somatic MMR alterations and integrated tumor–germline review should be considered. This framework is intended as a practical clinical aid and has not been prospectively validated as a bladder cancer-specific testing algorithm.

## What changes after lynch syndrome is confirmed?

A confirmed Lynch syndrome diagnosis has important implications for long-term cancer prevention, but much less certain implications for treatment of the presenting bladder cancer (1, 22, 23). For the index patient, confirmation of Lynch syndrome places the patient within established hereditary cancer surveillance and prevention pathways, most clearly for colorectal cancer, with management of other associated cancer risks adapted to the affected gene and individual clinical context (23).

Urologic surveillance is considerably less well established. In a systematic review of seven studies comprising 1,564 Lynch syndrome carriers, urothelial surveillance detected 11 cancers while 15 were missed (24). Urinalysis and urine cytology had calculated sensitivities of only 11% and 30%, respectively (24). Urine MSI testing showed promising performance, but the evidence came from a single prospective study of 81 individuals with *MSH2* variants and remains insufficient to support routine implementation. Current EAU guidance similarly states that optimal long-term screening and follow-up of Lynch syndrome carriers, with or without previous UTUC, remain undefined (8).

Evidence that germline Lynch syndrome should alter oncologic treatment of the bladder tumor itself remains limited. Current EAU bladder cancer guidance does not use Lynch syndrome status to define TURBT, intravesical treatment, radical cystectomy, or bladder-preserving therapy according to a separate oncologic pathway (9). In a recent single-institution series of 27 patients with Lynch syndrome-associated urothelial carcinoma, 70% had upper-tract disease and 48% experienced recurrence during a median follow-up of 92 months; the cohort was neither bladder-specific nor designed to compare treatment strategies (22). Available evidence therefore does not support modifying standard stage- and risk-adapted treatment of localized bladder cancer solely because Lynch syndrome has been identified (9, 22, 25).

Germline status may nevertheless affect surgical planning in selected patients. In women undergoing radical cystectomy who have an increased hereditary ovarian cancer risk, including those with Lynch syndrome, the EAU advises salpingo-oophorectomy after completion of childbearing and in women older than 40 years (9, 25).

In advanced urothelial carcinoma, germline Lynch syndrome should be distinguished from the tumor dMMR/MSI-H phenotype. In a multicenter cohort of 411 patients treated with immune checkpoint inhibitor monotherapy, MSI-H was associated with numerically longer overall survival in some treatment settings, but the associations were not consistently statistically significant (26). A recent single-institution series of 24 patients with advanced dMMR/MSI-H UTUC reported an objective response rate of 83% with single-agent immune checkpoint blockade; 16 patients had confirmed Lynch syndrome, but the small retrospective cohort and exclusive upper-tract population limit extrapolation to bladder cancer (27). These findings support further study of dMMR/MSI-H as a therapeutic biomarker in urothelial carcinoma, but do not establish germline Lynch syndrome itself as an independent biomarker for systemic treatment selection in bladder cancer (26, 27).

The familial implications of a germline diagnosis are more direct. Identification of the familial pathogenic variant enables cascade testing of at-risk relatives, allowing unaffected carriers to enter appropriate Lynch syndrome surveillance and prevention programs (23, 28). Thus, when Lynch syndrome is identified through a bladder cancer diagnosis, its main clinical impact may extend beyond treatment of the index tumor to long-term cancer prevention for the patient and hereditary risk assessment for the family.

## Discussion

Existing literature has largely approached Lynch syndrome and urothelial cancer from two directions: germline testing across urothelial carcinoma broadly, and urologic surveillance in patients already known to have Lynch syndrome (10, 24). The reverse clinical problem: whether a diagnosis of bladder cancer should prompt evaluation for previously unrecognized Lynch syndrome, remains less well defined. The available evidence supports integrating clinical history with tumor molecular findings rather than transferring UTUC criteria directly to bladder cancer, with individual findings carrying different weight according to the strength and bladder-specificity of the supporting evidence (10, 11, 16, 18). For now, we favor a selective approach over universal tumor or germline testing in unselected bladder cancer populations, pending prospective validation.

The immediate research need is prospective validation of this approach in unselected bladder cancer populations. Kamau et al. identified Lynch syndrome in 0.66% and 0.85% of two unselected urinary tract cancer cohorts, but both included tumors of the bladder and upper urinary tract and therefore cannot establish the prevalence of Lynch syndrome in bladder cancer alone (29). Bladder-specific studies should compare universal with selective testing, quantify the incremental yield of combining clinical selection with MMR IHC, MSI, and tumor sequencing, and determine how often dMMR/MSI-H bladder tumors reflect germline Lynch syndrome rather than somatic MMR deficiency (16, 29).

A separate question is whether Lynch-associated bladder cancer has a distinct natural history or treatment response. The available clinical series remain small and frequently combine bladder and upper-tract disease; the recent cohort of 27 Lynch-associated urothelial cancers, for example, included 70% UTUC, limiting bladder-specific conclusions (22). Future studies should evaluate germline Lynch status and tumor dMMR/MSI-H separately in relation to recurrence, progression, response to BCG and bladder-preserving treatment, systemic therapy, and long-term urothelial outcomes. Effective post-diagnosis surveillance also remains unresolved, given the limited performance of currently studied urinary screening methods (22, 24).

Until these data are available, the hierarchy proposed in this review provides a practical way to interpret the existing evidence without converting incompletely validated associations into formal testing criteria. Its purpose is not to replace established hereditary cancer pathways, but to reduce missed opportunities for Lynch syndrome evaluation when bladder cancer is the presentation that brings the patient to urologic care. Recognition in this setting may have consequences extending well beyond the index tumor, including lifelong cancer prevention and hereditary risk assessment within the family.
